# Repetitive Cerulein-Induced Chronic Pancreatitis in Growing Pigs—A Pilot Study

**DOI:** 10.3390/ijms24097715

**Published:** 2023-04-23

**Authors:** Ewa Tomaszewska, Małgorzata Świątkiewicz, Siemowit Muszyński, Janine Donaldson, Katarzyna Ropka-Molik, Marcin B. Arciszewski, Maciej Murawski, Tomasz Schwarz, Piotr Dobrowolski, Sylwia Szymańczyk, Sławomir Dresler, Joanna Bonior

**Affiliations:** 1 Department of Animal Physiology, Faculty of Veterinary Medicine, University of Life Sciences in Lublin, 20-950 Lublin, Poland; sylwia.szymanczyk@up.lublin.pl; 2 Department of Animal Nutrition and Feed Science, National Research Institute of Animal Production, 32-083 Balice, Poland; malgorzata.swiatkiewicz@iz.edu.pl; 3 Department of Biophysics, Faculty of Environmental Biology, University of Life Sciences in Lublin, 20-950 Lublin, Poland; siemowit.muszynski@up.lublin.pl; 4 School of Physiology, Faculty of Health Sciences, University of the Witwatersrand, Parktown, Johannesburg 2193, South Africa; janine.donaldson@wits.ac.za; 5 Department of Animal Molecular Biology, National Research Institute of Animal Production, 32-083 Balice, Poland; katarzyna.ropka@iz.edu.pl; 6 Department of Animal Anatomy and Histology, Faculty of Veterinary Medicine, University of Life Sciences in Lublin, 20-950 Lublin, Poland; mb.arciszewski@wp.pl; 7 Department of Animal Nutrition, Biotechnology and Fisheries, Faculty of Animal Science, University of Agriculture in Kraków, 30-059 Kraków, Poland; rzmmuraw@cyf-kr.edu.pl; 8 Department of Animal Genetics, Breeding and Ethology, Faculty of Animal Science, University of Agriculture in Kraków, 30-059 Kraków, Poland; rzschwar@cyf-kr.edu.pl; 9 Department of Functional Anatomy and Cytobiology, Faculty of Biology and Biotechnology, Maria Curie-Sklodowska University, 20-033 Lublin, Poland; piotr.dobrowolski@umcs.lublin.pl; 10 Department of Analytical Chemistry, Medical University of Lublin, 20-059 Lublin, Poland; 11 Department of Plant Physiology and Biophysics, Faculty of Biology and Biotechnology, Maria Curie-Skłodowska University, 20-033 Lublin, Poland; 12 Department of Medical Physiology, Chair of Biomedical Sciences, Institute of Physiotherapy, Faculty of Health Sciences, Jagiellonian University Medical College, 31-126 Kraków, Poland; joanna.bonior@uj.edu.pl

**Keywords:** pig, chronic pancreatitis, cerulein, cytokines, junction proteins

## Abstract

Chronic pancreatitis (CP) is an irreversible and progressive inflammatory disease. Knowledge on the development and progression of CP is limited. The goal of the study was to define the serum profile of pro-inflammatory cytokines and the cell antioxidant defense system (superoxidase dismutase—SOD, and reduced glutathione—GSH) over time in a cerulein-induced CP model and explore the impact of these changes on selected cytokines in the intestinal mucosa and pancreatic tissue, as well as on selected serum biochemical parameters. The mRNA expression of *CLDN1* and *CDH1* genes, and levels of Claudin-1 and E-cadherin, proteins of gut barrier, in the intestinal mucosa were determined via western blot analysis. The study showed moderate pathomorphological changes in the pigs’ pancreas 43 days after the last cerulein injection. Blood serum levels of interleukin (IL)-1-beta, IL-6, tumor necrosis factor alpha (TNF-alpha), C-reactive protein (CRP), lactate dehydrogenase (LDH), gamma-glutamyl transpeptidase (GGTP), SOD and GSH were increased following cerulein injections. IL-1-beta, IL-6, TNF-alpha and GSH were also increased in jejunal mucosa and pancreatic tissue. In duodenum, decreased mRNA expression of *CDH1* and level of E-cadherin and increased D-lactate, an indicator of leaky gut, indicating an inflammatory state, were observed. Based on the current results, we can conclude that repetitive cerulein injections in growing pigs not only led to CP over time, but also induced inflammation in the intestine. As a result of the inflammation, the intestinal barrier was impaired.

## 1. Introduction

Chronic pancreatitis (CP) is an irreversible and progressive inflammatory disease, the diagnosis of which is problematic due to the long, asymptomatic period. CP affects both exocrine and endocrine pancreas function, resulting in pancreatic insufficiency, when more than 90% of the pancreas is damaged [1,2,3]. Despite the pathomorphological changes that occur during acute pancreatitis (AP) being well-characterized, knowledge regarding the pathomorphological changes in patients with CP during the early asymptomatic period is lacking, which, together with the fact that tracking the patients’ symptoms is nearly impossible, makes diagnosis difficult. CP has different signs depending on the severity of the disease; however, it is usually associated with steatorrhea, weight loss and even multiple organ dysfunction [4]. The etiology of CP is multifactorial, with the average age of diagnosis in humans being between 35 and 55 years old, when CP is already advanced, with severe pancreatic pathomorphological alterations, such as calcification, observed [5,6]. Knowledge on the development and progression of CP is limited, and the diagnosis of early CP is controversial and challenging [7,8]. For this reason, the best way to study all CP symptoms in detail is to carry out studies on animal models with induced CP. Most models, mainly of CP, require further characterization to determine all pathophysiological changes [7,9,10].

Although cerulein-induced CP is frequently used on other animal models, rodent models are primarily used, since rodent studies are less complex and time-consuming [11,12]. The pig model of CP is the most suitable model to track inflammatory reactions over time, which mimic the changes observed during the human inflammatory process [11,12]. Moreover, the choice of the pig as an experimental animal model is justified because the physiology of the pig is comparable to that of humans and the pig’s pancreas is very similar to the human pancreas, such as in its topographical location, including a pancreatic duct which drains into the duodenum, similar cellular components (acinar, ductal, stellate and endocrine cells), as well as in exerting both exocrine and endocrine functions. The pancreas of the pig is not able to regenerate as has been previously observed in rats [13]. Thus, the pig is the primary species of interest [14,15]. Moreover, cerulein-induced CP in growing pigs is a minimally invasive and non-surgical method leading to a progressive systemic inflammation, marked by rapid progression, resulting in the weakening of the organism.

We hypothesized that repetitive induction of acute cerulein-induced pancreatitis leads to CP in pigs over time.

Studies on CP are rarely presented, and none of the existing CP studies involve USG imaging, since visualization of the pancreas in animals via in vivo imaging and ex vivo dissection is difficult. Therefore, in this preclinical study, the USG imaging method was used to investigate cerulein-induced CP, as well as changes in the serum pro-inflammatory profile during CP development.

The primary aims of the study were to define the profile of serum pro-inflammatory cytokines and the cell antioxidant defense system (SOD and GSH) over time in a cerulein-induced CP model, and to explore the impact of these changes on selected cytokines in the intestinal mucosa and pancreatic tissue, as well as on selected serum biochemical parameters. The mRNA expression of *CLDN1* and *CDH1* genes, encoding tight junction proteins, Claudin-1 and adherens junction protein E-cadherin, respectively, and protein levels of Claudin-1 and E-cadherin in the intestinal mucosa were determined via Western blot analysis.

## 2. Results

### 2.1. Body Weight and Daily Weight Gain

**Figure 1:**
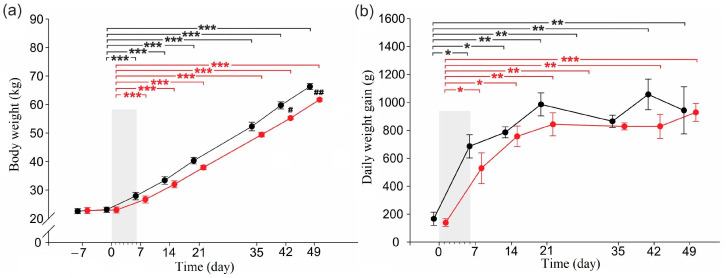
Changes in (**a**) body weight, (**b**) and daily weight gain of pigs during the experimental period. Shadowed area shows the 6-day period of daily cerulein injections (1 µg/kg b.w./day) in the CER-pig group. Black: the control group (C-pigs); red: the cerulein-injected group (CER-pigs). Data are presented as mean ± SE. Statistical significance: * *p* < 0.05; ** *p* < 0.01; *** *p* < 0.001 (compared to day 0). Statistical significance: ^#^ *p* < 0.05; ^##^ *p* < 0.01 (between groups).

There were no significant differences in mean body weight between groups at the start of the adaptation period (day −7) or at the end of the adaptation period (day 0) (Figure 1a). The body weight of all pigs increased significantly over the 49-day experimental period; however, the CER-pigs weighed significantly less than the C-pigs on days 42 and 49. The daily weight gain was comparable in both groups (Figure 1b).

### 2.2. Serum Nutritional and Other Basal Biochemical Parameters

**Figure 2:**
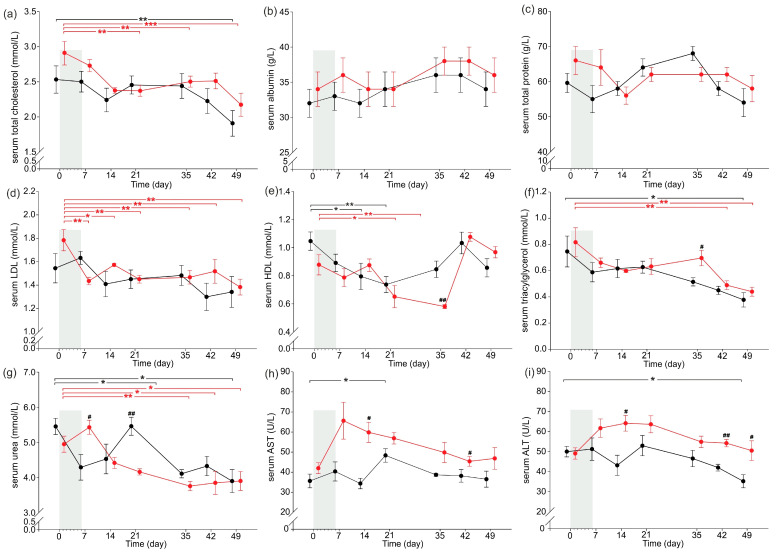
Changes in serum nutritional parameters (**a**) TCHOL and (**b**) ALB and other biochemical parameters: (**c**) TPRT, (**d**) LDL, (**e**) HDL, (**f**) TG, (**g**) UREA, (**h**) AST, and (**i**) ALT of pigs during the experimental period. Shadowed area shows the 6-day period of daily cerulein injections (1 µg/kg b.w./day) in CER-pig group. Black: the control group (C-Pigs); red: the cerulein-injected group (CER-pigs). Data are presented as mean ± SE. Statistical significance: * *p* < 0.05; ** *p* < 0.01; *** *p* < 0.001 (compared to day 0). Statistical significance: ^#^ *p* < 0.05; ^##^ *p* < 0.01 (between groups). LDL—low-density lipoprotein, HDL—high-density lipoprotein, AST—aspartate aminotransferase, AL—alanine aminotransferase.

Serum nutritional parameters are presented in Figure 2a,b, while other basal biochemical parameters are presented in Figure 2c–i. Serum total cholesterol (TCHOL) concentrations were significantly decreased on day 21, 28 and 49 compared to day 0 in CER-pigs, while in C-pigs, serum TCHOL was only significantly lower than that observed on day 0 at the end of the experiment (day 49). Serum LDL concentrations were significantly higher in CER-pigs compared to C-pigs on day 0, decreased significantly following cerulein injections (day 7) and were still significantly lower at the end of the experimental period compared to day 0. Serum HDL concentrations were decreased on day 21 and on day 28 compared to day 0 in the CER-pigs, while in the C-pigs, a decrease was noted on day 14 and day 21 compared to day 0. The concentration of serum TG was significantly decreased on day 42 and day 49 compared to day 0 in the CER-pigs (5 and 6 weeks, respectively, after completion of the cerulein injections). Serum TG concentrations were also significantly lowered at the end of the experimental period (day 49) compared to day 0 in the C-pigs; however, three weeks from completion of the cerulein injections, the TG concentration was significantly higher in CER-pigs compared to that in C-pigs (day 28). 

Serum urea concentrations decreased three weeks following completion of the cerulein injections (day 28) in the CER-pigs and was still lowered until the end of the experiment (day 42 and 49) compared to the initial values (day 0). C-pigs displayed decreased serum urea concentrations in the 4th and 6th weeks of the experimental period (day 28 and 49) compared to day 0. Serum urea concentrations in CER-pigs were significantly higher on day 7 and lower on day 21 compared to that observed in C-pigs on these days. AST activity increased significantly in C-pigs on day 21 compared to that observed on day 0; however, AST activity was significantly higher in CER-pigs on day 21 and day 42 compared to that observed in C-pigs on the same days. Significantly decreased ALT activity was observed in C-pigs on day 49, while CER-pigs displayed significantly increased ALT activity on days 14, 42 and 49 compared to that of the C-pigs. No other changes were observed.

### 2.3. Inflammatory Parameters

**Figure 3:**
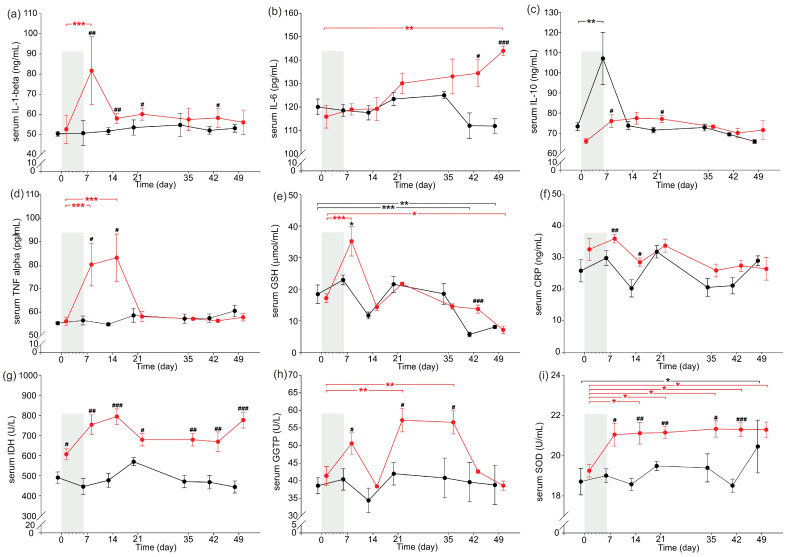
Changes in serum cytokines and inflammatory parameters: (**a**) IL-1-beta, (**b**) IL-6, (**c**) IL-10, (**d**) TNF-alfa, (**e**) GSH, (**f**) CRP, (**g**) LHD, (**h**) GGTP, and (**i**) SOD of pigs during the experimental period. Shadowed area shows the 6-day period of daily cerulein injections (1 µg/kg b.w./day) in CER-pig group. Black: the control group (C-pigs); red: the cerulein-injected group (CER-pigs). Data are presented as mean ± SE. Statistical significance: * *p* < 0.05; ** *p* < 0.01; *** *p* < 0.001 (compared to day 0). Statistical significance: ^#^ *p* < 0.05; ^##^ *p* < 0.01; ^###^ *p* < 0.001 (between groups). IL-1-beta—interleukin 1 beta, IL-6—interleukin 6, IL-10—interleukin 10, TNF alpha—tumor necrosis factor alpha, GSH—reduced glutathione, CRP—C-reactive protein, LDH—lactate dehydrogenase, GGTP—gamma-glutamyl transpeptidase, SOD—total superoxidase dismutase.

Inflammatory parameters are presented in Figure 3. Serum IL-1-beta concentrations increased significantly just after completion of the cerulein injections (day 7) compared to that observed on day 0 in CER-pigs, with serum concentrations being significantly higher in CER-pigs on days 7, 14, 21 and 42 compared to those observed in C-pigs on these days (Figure 1a). CER-pigs had significantly increased serum IL-6 concentrations 6 weeks after completion of the cerulein injections (at the end of the experiment, day 49), and the serum IL-6 concentration was also significantly higher compared to that of the C-pigs on the same day. Serum IL-10 concentrations were significantly increased in C-pigs on day 7 compared to day 0 and were significantly higher than the IL-10 concentrations observed in CER-pigs at the same time. Moreover, the serum IL-10 concentrations in CER-pigs were significantly higher two weeks after finishing the cerulein injections (day 21) compared to those of the C-pigs. Serum TNF-alpha concentrations were significantly increased in CER-pigs on day 7 and day 14 compared to day 0 and were significantly higher than those noted in C-pigs on the same days.

Serum GSH concentrations increased significantly just after completion of the cerulein injections (day 7) in CER-pigs and were significantly lower at the end of the experiment (day 49) compared to day 0 (before cerulein injections), while in the C-pigs, serum GSH concentrations were significantly decreased on day 42 and day 49 compared to day 0. Moreover, GSH concentrations were significantly higher in CER-pigs compared to C-pigs on day 7 and day 42. CER-pigs had significantly increased serum CRP concentrations just after finishing the cerulein injections on day 7 and day 14 compared to those noted in the C-pigs. Serum LDH activity was higher in CER-pigs compared to C-pigs throughout the whole study period. Serum GGTP activity was significantly increased on day 21 and day 28 in CER-pigs compared to day 0, and was significantly higher in CER-pigs compared to C-pigs on days 7, 21 and 28. Serum SOD activity increased significantly one week after cerulein injections in CER-pigs and remained significantly increased compared to day 0 throughout the next 5 weeks (days 7–49). C-pigs displayed significantly increased SOD activity at the end of the study (on day 49) compared to day 0. Moreover, SOD activity was significantly higher in CER-pigs compared to C-pigs on days 7–42. 

### 2.4. Pathomorphological Description of Pancreatic Samples

The histological sections of the pancreas from C-pigs and CER-pigs were evaluated by two independent histologists, each with more than 20 years of experience. Both histologists were blinded to the group coding. 

**Figure 4:**
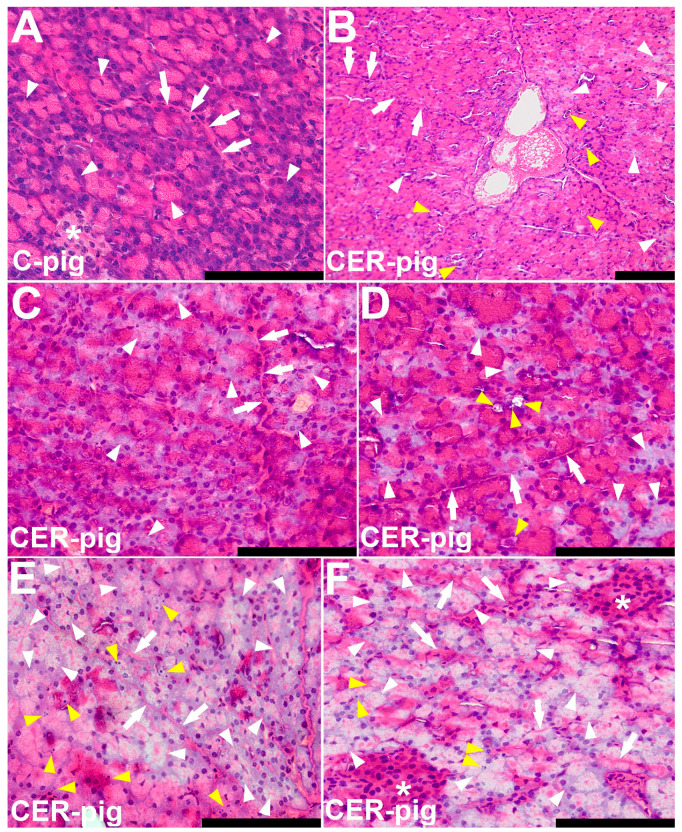
Pathomorphological changes observed in pig pancreas sections. Representative hematoxylin and eosin (H&E)-stained sections of pancreas: (**A**) control group with normal (healthy) pancreas. Acinar cells stained blue at their base due to the high content of RNA and the presence of nuclei, and pink at their apex (lumenal aspect) due to a high content of zymogen granules with proteins (digestive enzymes); arrowheads show acinar cells forming acinar glands of exocrine pancreas; asterisk marks a small pancreatic islet (small cells, pale cytoplasm; islets of Langerhans) in the more abundant and darker acinar tissue where the islet cells are smaller and have paler cytoplasm than the surrounding acinar cells; arrows show an intralobular duct with a modest collagenous wall; the lumen of the small duct contains homogenous pink staining protein-rich pancreatic juice. (**B**–**F**) are representative photographs of the pancreas from pigs with cerulein-induced pancreatitis, as the inflammation is not evenly distributed in the pancreatic parenchyma. The images show the characteristics of the pancreatitis observed. Yellow arrowheads show cellular necrosis (**B**,**D**) or apoptosis (**E**,**F**); white arrowheads show degeneration of acinar cells, pale cytoplasm with no zymogen granules, acinar edema; asterisks mark a darker pancreatic islet (small cells, pale cytoplasm—islets of Langerhans) in the more abundant pale, swollen and degenerative acinar tissue (**F**); arrows show intralobular ducts with degradation of ductal epithelium (**D**,**E**) or with lymphatic infiltration (**F**). All scale bars represent 100 µm.

Pathomorphological changes in the pancreas sections are presented in Figure 4. Multiple signs of pancreatitis, ranging from mild to moderate and acute, were observed in the pancreatic parenchyma of CER-pigs. The symptoms were related to the exogenous part of the pancreatic parenchyma. The most prominent features were cellular edema with complete atrophy of the luminal zymogen granulation, most likely causing further disruption of the adjacent acinar structure, acinar atrophy, and cellular necrosis, observed as areas of pale or no eosin staining. Disturbed acinar and ductal structure were noted in the normally stained parenchyma. Several specimens from CER-pigs also showed areas of increased apoptosis. Fibrosis and coagulative necrosis were observed in the acute pancreatitis stages. However, these were rare. In two cases, there was evidence of hemorrhage. There was no evidence of fatty necrosis and no evidence of lymphatic infiltration.

### 2.5. Pathomorphological Description of Pancreas Based on USG Examination

**Figure 5:**
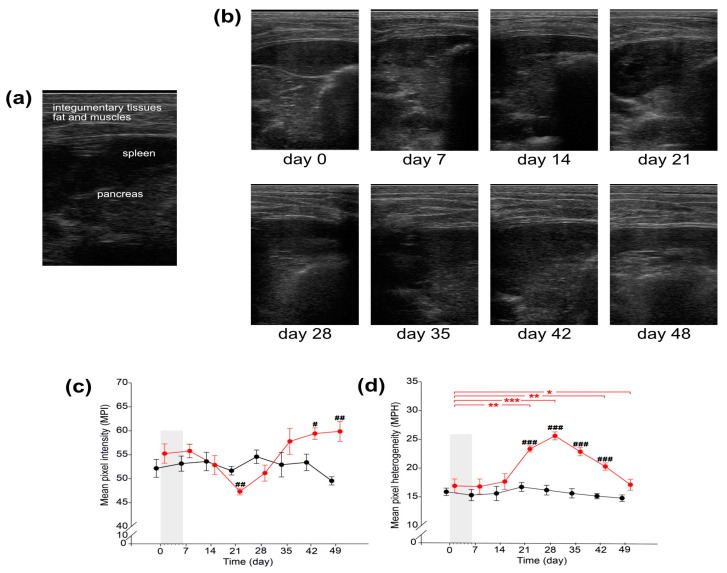
USG pancreas examination. (**a**) USG probe placement allowed visualization of a part of the pancreas, situated behind (underneath in the scan) the spleen, well-visible in the foreground straight under integumentary tissues. (**b**) Representative USG images on the timeline to show changes in pancreas echotexture after cerulein treatment. Quantitative analysis of USG images: (**c**) mean pixel intensity (MPI); (**d**) mean pixel heterogeneity (MPH). Black: the control group (C-pigs); red: the cerulein-injected group (CER-pigs). Data are presented as mean ± SE. Statistical significance: * *p* < 0.05; ** *p* < 0.01; *** *p* < 0.001 (compared to day 0). Statistical significance: ^#^ *p* < 0.05; ^##^ *p* < 0.01; ^###^ *p* < 0.001 (between groups).

USG analysis showed inflammation in the pancreas of CER-pigs which started just after the completion of the cerulein injections, on day 7 (24 h after finishing cerulein injections), and persisted until the end of the experiment, when the pancreatic parenchyma in CER-pigs showed features of chronic inflammation, with signs of peripancreatic inflammation (Figure 5b). From day 14, echogenicity was mixed (intensified to reduced), and from day 28, noticeable small hyperechogenic foci with visible irregular fibrosis, and with suspected interstitial calcification, were observed. Pancreatic tissue in C-pigs was uniformly echogenic and was similar in echotexture to the liver, without signs of hypertrophy and inflammation, although one pig had slight heterogeneity in echogenicity. 

These observations were confirmed via the analyses of mean pixel intensity (MPI) and mean pixel heterogeneity (MPH) (Figure 5c,d). Although MPI detected over time was not different from that observed on day 0, when cerulein injections started, it decreased significantly on day 21 and then increased, reaching values that were significantly higher than that observed in C-pigs on days 42 and 49. MPH was significantly increased in CER-pigs 15 days after finishing cerulein injections (on day 21), and the pancreas was still heterogenous on days 28, 35, and 42 compared to that observed on day 0; in addition, MPH was significantly increased compared to that observed in the C-pigs on the aforementioned days. 

### 2.6. Interleukins and Reduced GSH in Tissues

**Figure 6:**
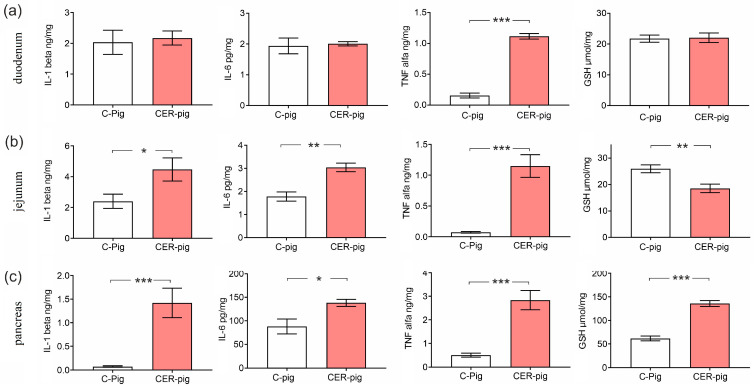
Changes in interleukins and reduced GSH in (**a**) duodenal and (**b**) jejunal mucosa, and (**c**) pancreatic tissue. Data are presented as mean ±SE. Statistical significance: * *p* < 0.05; ** *p* < 0.01; *** *p* < 0.001 (Tukey’s HSD test). IL-1-beta—interleukin 1 beta, IL-6—interleukin 6, TNF alpha—tumor necrosis factor alpha, GSH—reduced glutathione.

The concentration of TNF-alpha in the duodenal mucosa was significantly higher in CER-pigs compared to that of the C-pigs. The concentrations of IL-1-beta, IL-6, and TNF-alpha were significantly higher in the jejunal mucosa and pancreas of CER-pigs compared to that observed in C-pigs. The concentration of GSH in the jejunal mucosa was significantly lower in CER-pigs compared to C-pigs, while it was significantly higher in the pancreatic tissue of CER-pigs compared to C-pigs. No other changes were observed (Figure 6).

### 2.7. Expression of Intestinal Barrier Proteins and Their Genes and Intestinal Barrier Integrity

**Figure 7:**
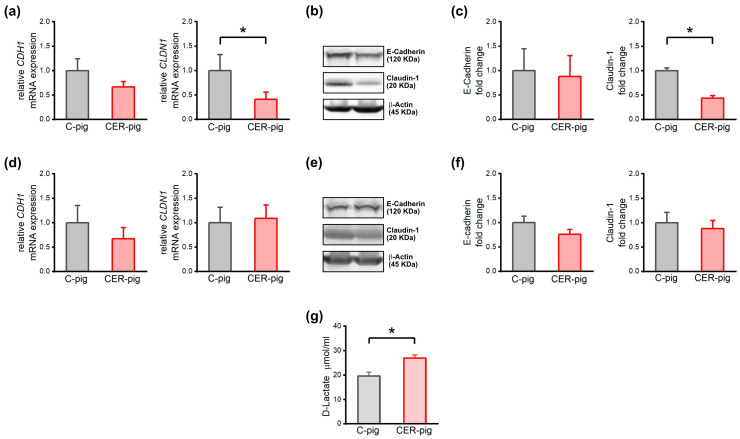
Effect of cerulein-induced chronic pancreatitis on intestinal barrier integrity. Quantitative real-time PCR analysis of mRNA expression of *CDH1* and *CLDN1* genes, encoding E-cadherin and Claudin-1 protein, respectively, in (**a**) duodenum and (**d**) jejunum. Due to the exponential nature of the mRNA expression calculated using the -ddCT method, the geometric means with standard errors are presented. Western blot analysis of E-cadherin and Claudin-1 in (**b**) duodenum and (**e**) jejunum, β-actin was used as loading control. (**c**) Protein expression level of Claudin-1 and E-cadherin in (**c**) duodenum and (**f**) jejunum; The protein levels are normalized to the corresponding β-actin levels and expressed as the fold change relative to the amount present in C-pigs; Uncropped original western blot membranes of E-Cadherin and Caludin-1 can be found in Supplementary Figure S1; (**g**) blood serum concentrations of D-lactate, an indicator of the permeability of the intestinal mucous membrane. Error bars represent standard errors. * *p* < 0.05. (Tukey’s HSD test).

Duodenal expression of the *CLDN1* mRNA and Claudin-1 protein was significantly decreased in CER-pigs compared to C-pigs at the end of the experimental period, on day 49 (Figure 7a–c). No other changes in mRNA transcript levels of tight and adherens junction proteins were noted (Figure 7). Blood serum concentrations of D-lactate were significantly higher in CER-pigs compared to C-pigs (Figure 7g).

## 3. Discussion

As previously mentioned, pancreatitis, one of the most common problems in gastroenterology, is linked to serious complications and high patient mortality [16,17,18,19]. Cerulein, a decapeptide, is an analogue of CCK and a potent stimulant of the musculature of the gallbladder and the intestine, as well as a powerful stimulant of pancreatic secretion and moderate stimulant of gastric secretion [20,21,22,23]. Cerulein is used in rodent and non-rodent studies to induce secretory effects or pancreatic injury in a time-, species-, dose- and rout-dependent manner [20,24,25,26,27]. This classic model, established in 1977 by Lampel and Kern [28], is well-established and has been shown to induce pancreatitis, with biochemical, pathophysiological and structural similarities to that of human pancreatitis [29,30]. For this reason, it is the most frequently used and widely known non-invasive, experimental model of relevance for investigation of the pathogenesis of both acute and CP, which can be controlled through the appropriate dosage and frequency of injection of cerulein [7,31]. There is a clear dose–response relationship between the structural and biochemical changes in the pancreas in response to cerulein administration, which also depends on the route of administration, as mentioned above [20,32,33]. Repetitive cerulein-induced CP is a classic animal model of CP which is easy to establish and applicable in many species due to the rapid development of CP [34,35]. However, the severity of cerulein-induced CP depends on the number of repeated applications [12,36]. In general, in many cerulein-induced CP animal models, the animals are in visible pain during the development of CP, which correlates with the pathomorphological changes and increased sensory neuron activity and is comparable to that noted in humans [12,37]. Even though pigs develop CP with a similar histopathology to human CP, no specific pain is involved [12]. These observations were consistent with the current study, since there were no changes observed in pig behavior such as inactivity, arched back or anorexia observed in pigs injected repetitively with cerulein in 24 h intervals, at a dose of 1 µg/kg b.w. A gradual decrease in BW was observed, which was statistically significant on days 42 and 49 compared to day 0; thus, the current study could not continue past the 49 days, due to the ethical restrictions related to a decrease in BW. For this reason, we are still unsure what could have happened over time if the study had continued. Under normal pathological conditions of CP, one could expect a further decrease in BW; however, in the case of cerulein-induced CP, a recovery in BW has been confirmed by different studies [34,38]; thus, an improvement in BW could be expected, though further studies are needed to confirm this possibility. On the other hand, it is not known whether the pathological alterations observed in the pancreas of the CER-pigs were reversible or not. Cerulein-induced pancreatitis is characterized by a moderate onset of injury in the pancreas lasting between 24 and 48 h [36,39]. Moderate CP was seemingly achieved in the pigs in the current study, since they did not seem to be in any pain or suffer any maldigestion or steatorrhea which commonly occurs during extensive pancreatic injury, which leads to a 10% decrease in lipase activity [40]. Additionally, repetitive cerulein-induced CP is not associated with exocrine and endocrine dysfunction according to the new mechanistic CP definition [41,42]. Our results were in line with this characteristic because the pigs that received repetitive cerulein injections and developed pancreatitis did not present any changes in serum insulin over time, nor in serum lipase or amylase activity until the end of the study. 

Moreover, the method used in the current study to induce pancreatitis mimics that which occurs in humans, where clinical observations have shown that repetitive episodes of short acute pancreatitis, irrespective of origin, lead to organ damage, resulting in CP [36,43]. In the current study, repetitive cerulein injections triggered short-lasting acute pancreatitis (AP), which ultimately led to the development of CP, the extent of which depends on the intervals between injections and the dose of cerulein used. The current study showed that 43 days after the last (6th) cerulein injection, moderate pathomorphological changes were observed in the pigs’ pancreas. One of the limitations of the current study was the lack of biopsies of pancreatic tissue over time during the experiment, which would have allowed us to track the development of the pathomorphological changes. On the other hand, we made use of USG, which is a less invasive method for the assessment of pancreatic pathology, including pancreatitis [44]. This method is commonly used in pigs to assess the composition of the musculature [45,46]. In the current study, USG examination was used to assess cerulein-induced pancreatic alterations. Textural changes with slight calcifications foci, which are frequently observed in humans with CP [47], were observed in the pancreas of CER-pigs on day 35 and day 42 and were related to changes in biochemical parameters such as IL-6, GGTP and SOD, which peaked at the same time. These characteristics of CP observed in the CER-pigs, including calcification and alteration in gland echo texture, were in line with the Cambridge classification of CP in 1984 [48].

The pathogenesis of pancreatitis is well-understood. It is an inflammatory condition which is initiated and/or sustained by a disturbance in the regulation of enzyme secretion by pancreatic acinar cells [49,50,51]. However, chronic cerulein-induced pancreatitis is initiated via a trypsinogen activation-independent inflammatory response, where cerulein stimulates acinar cells via the cholecystokinin receptor (G protein-coupled receptor) and the protein kinase C (PKC) pathway or the phosphatidylinositol 3-kinase (PI3K) pathway and the release of intracellular calcium, which in turn activates nuclear factor kappa B (NF-kB) [52]. The release of NF-kB stimulates pro-inflammatory target genes such as that of TNF-alpha and monocyte chemotactic protein-1 (a chemokine necessary for the influx of inflammatory macrophages into the pancreas), leading to the synthesis of a host of pro-inflammatory factors that have the potential to drive various aspects of pancreatic inflammation, apoptosis, cell differentiation and proliferation [52,53]. TNF-alpha, secreted mainly by macrophages during CP, results in apoptotic or necrotic acinar cell injury and plays a critical role in the development of experimental pancreatic inflammation [52,54]. In addition to TNF-alpha, other pro-inflammatory cytokines also play a role in the development of pancreatic inflammation. Pro- and anti-inflammatory cytokine, IL-6, enhances inflammation mainly via pathological T helper-type 17 cells and stimulates the synthesis of CRP in the liver [55,56]. IL-1-beta induces neutrophil infiltration into the inflammatory area and induces other pro-inflammatory cytokines and chemokines [57]. In a normal physiological state, their concentration in plasma is low and there is an equilibrium between pro- and anti-inflammatory cytokines, whereas during an inflammatory stage, they play an auto-, para- and endocrine function, leading to systemic inflammation. Pro-inflammatory cytokines are released by the pancreas, the endothelium of blood vessels and by tissue macrophages [58]. The most potent anti-inflammatory cytokine is IL-10, which inhibits the synthesis of Th1 cells and the release of pro-inflammatory cytokines and decreases the activity of macrophages and monocytes. Serum IL-1β, IL-6 and IL-10 concentrations have been shown to increase after 6 to 12 h of severe acute pancreatitis in pigs [59]. 

In the current study, the first blood collection after the cerulein injections was performed within 24 h. One should remember that the cerulein-induced CP model is characterized by repetitive acute pancreatitis, induced by each cerulein injection, which eventually leads to the development of CP over time, which was confirmed in the current study by the pathomorphological images of the pigs’ pancreas. The current study assessed several cytokines that have not yet been studied in an experimental repetitive cerulein-induced CP pig model. During the first inflammatory response induced in the current study, an increase in serum IL-1-beta was observed just after completion of the repetitive cerulein injections (day 7). Serum TNF-alpha also increased simultaneously (days 7 and 14) and an increase in serum IL-6 was observed at the end of the study period (on day 49). On the other hand, IL-6, which is considered an anti-inflammatory cytokine, prevented the synthesis of IL-1-beta and TNF-alpha (the concentrations of which were comparable with the control values on day 49). Further studies are needed to check whether IL-6 is anti-inflammatory in the pathogenesis of experimental repetitive cerulein-induced CP in the pig model. 

In the current study, other indicators of inflammation were also assessed. A useful marker such as CRP, one of the acute phase proteins [60], was significantly higher than the control values on day 7, indicating inflammation. Serum LDH, which serves as a prognostic indicator for the evaluation of the severity of pancreatitis [61,62], was significantly elevated in CER-pigs throughout the whole study period. Elevated GGTP has also been noted in many different clinical conditions including pancreatic diseases [63]. Serum GGTP was significantly increased in CER-pigs on days 7, 21 and 28 in the current study. Serum ALT and AST are also useful parameters in the examination of pancreatitis [64]. Both parameters were significantly increased in CER-pigs on day 14 and day 49 in the current study, possibly indicating that acute inflammation occurred just after the cerulein injections, which eventually led to the development of CP [65] and other health problems over time which could be related to liver or muscle tissue [66]. 

Pancreatitis involves free radicals and their scavengers; thus, oxidative stress is important in the pathogenesis of pancreatic injury. SOD is a primary antioxidant enzyme present in three forms in mammals: extracellular, mitochondrial manganese, cytosolic/zinc. AP is characterized by oxygen-derived free radicals, the production of which decreases SOD concentrations [67]. The current time–course study on cerulein-induced CP in pigs showed that six repetitive cerulein injections, with 24 h intervals, resulted in a significant increase in serum SOD in relation to that observed prior to the cerulein injections and to that observed in the C-pigs. This result was in line with that observed in an in vivo rodent model study, lasting 24 weeks, which also showed an increase in *SOD* mRNA [67]. It is believed that SOD modulates chronic pancreatitis by suppressing apoptosis and promoting proliferation of acinar cells. Cerulein causes alterations in pancreatic acinar cells that lead to the formation of oxygen-derived radicals, an excess of which causes injury to other cells [68]. Although, the current SOD results were in agreement with those of Su et al. 2002 [67], they are not in agreement with results presented by Zheng et al. 2020 [69], who reported a decrease in SOD with the progression of pancreatitis; however, they presented patients with AP of different severities. Although GSH, a major nonenzymatic antioxidant generated intracellularly, decreased in patients with pancreatitis [70], the current study showed an initial increase in GSH followed by decrease over time. 

IL-1 beta, IL-6, TNF-alpha and GSH were assessed in duodenal and jejunal mucosa, as well as in pancreatic tissue. The results obtained confirm the general observation that CP should be considered a systemic inflammation [4]. The decrease in jejunal GSH could indicate that the tissue was unable to remove free radicals, which then damaged cells over and above those of the pancreatic tissue.

Inflammatory processes in humans are characterized by the production of CRP by the liver, along with other acute phase proteins, which in turn results in inhibition of the synthesis of other proteins such as albumin or lipoproteins [71]. The current study confirmed this observation, as serum LDL and HDL were both decreased following cerulein injections. Serum TG and TCHOL decreased gradually in both C- and CER-pigs; however, serum TGs were significantly higher in CER-pigs on day 35, although ALB (the negative acute phase protein, the synthesis of which declines during inflammation) [72] and TPRT remained unchanged. Serum TG is also associated with the severity and prognosis of pancreatitis, including pancreatic necrosis [73]. Urea is considered an early predictor of the severity of pancreatitis [74,75]. CER-pigs showed a peak in serum UREA just after the repetitive cerulein injections; however, a peak was also observed in the C-pigs, the reason for which is unknown. There is no doubt that this should be clarified. The same relates to TCHOL, which decreased on day 49 in C-pigs, contrary to the TCHOL decrease observed in CER-pigs, which could be linked to pancreatic insufficiency [76]. TCHOL is one of the nutritional markers important for the determination of the degree of pancreatic insufficiency resulting from CP, and these markers are routinely performed in clinical practice [76]. 

The gut also plays a role in the pathogenesis of pancreatitis, which has been proven in humans who suffer from increased gut permeability, which correlates with pancreatic injury severity [77]. Pathomorphological alterations in intestinal mucosa are observed in patients with CP, mainly in the duodenum [78,79]. Claudin-1, a tight junction protein, which is also present in the human intestine [80], is up-regulated in intestinal inflammation [81] and plays a significant role in pancreatic cancer invasion; for this reason, it is useful as a biomarker during disease [82]. The cell adhesion protein, E-cadherin, also plays a pivotal role in tissue formation and homeostasis [83,84]. Previous studies have shown up-regulated E-cadherin expression in rats with chemically induced AP, while its degradation during CP has been noted and correlated with increased risk of cancer [85]. 

A decrease in Claudin-1 expression was observed in the CER-pigs in the current study, which is in agreement with data presented in humans. Additionally, a decrease in mRNA expression of the *CLDN1* gene was observed in the duodenum of CER-pigs. Moreover, D-lactate, an indicator of leaky gut, was increased in CER-pigs. This could indicate the possibility of the occurrence of carcinogenesis [86].

## 4. Materials and Methods

### 4.1. Ethics

The study was approved by the Local Ethics Committee for Animal Experiments in Cracow, Poland (No 377/2020). The methods were carried out in accordance with norms of the European Union law (Directive 2010/63/UE) on the protection of animals used for scientific purposes (received in Poland by Legislative Decree 266/2015). The experiment was carried out in compliance with the ARRIVE guidelines.

### 4.2. Animals and Experimental Design

**Figure 8:**
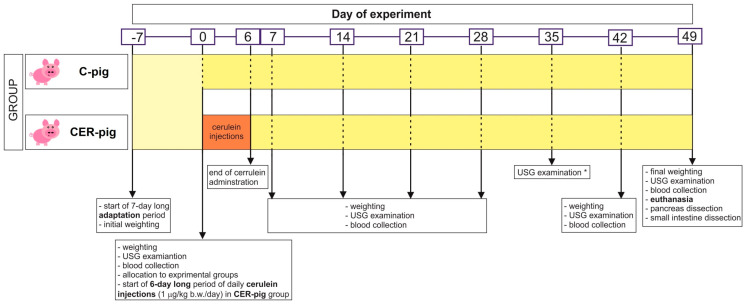
Experimental layout. The experiment lasted 56 days in total: 7 days of adaptation (−7 to 0), 6 days of cerulein injections were performed with 24 h intervals (between day 0 and day 6). Weighing, blood collection and USG pancreas examination were performed in one-week intervals; * on day 35, due to COVID-19 restrictions, only USG examination was performed. On day 49, after final measurements, the pigs were subjected to pharmacological euthanasia and pancreas and small intestine sections (duodenum, jejunum) were dissected.

The research was carried out on domestic pigs (PBZ breed) at the National Research Institute of Animal Production in Krakow. The study (Figure 8) involved 10 uncastrated boars, aged between 9 and 10 weeks [87], housed in individual metabolism cages. After a 7-day adaptation period (time period between points -7 and 0), the pigs were divided into two groups (point 0). Age-, sex- and weight-matched control pigs (C-pig) were used to track systemic inflammatory and pancreatic changes and compare them to pigs administered CER. After the adaptation period (the first 7 days; period between day -7 and 0), CER-pigs received intramuscular (i.m.) injections of cerulein (CAERULEIN, Sigma-Aldrich Merck KGaA, Darmstadt, Germany) at a dose of 1 µg/kg b.w./day, for 6 consecutive days, with 24 h intervals, to induce CP (period between day 1 and 6), while C-pigs were injected with vehicle (physiological saline) [11,88]. The animals were then under the constant supervision of qualified staff and a veterinarian for the next 6 weeks (period between day 7 and 49), during which body weight measurements and blood samples for analyses were taken every week, for a total of five times, except during the third week after stopping cerulein injections, due to COVID-19 restrictions, on day 28. Animals in both groups were fed the same feed mixture, intended for pigs of this age, which contained all nutrients at levels recommended by the NRC and according to Polish standards of pig nutrition [89,90]. The pigs had unlimited access to water from nipple drinkers. All pigs were weighed individually at the beginning of the adaptation period (day −7), before the start of the cerulein injections (day 0), at the end of the cerulein injections (day 7), and weekly for the next 6 weeks (day 14, 21, 35, 42), as well as prior to euthanasia on day 49. Blood samples were collected at the same time at which the pigs were weighed. Coagulated blood was centrifuged (1300× *g* for 10 min at 18 °C) to obtain serum, which was then collected, aliquoted and stored at −86 °C. 

At the end of the experimental period (on day 49; 43 days after the end of the cerulein injections), the pigs were fasted overnight and after final weighing, USG examination, and blood collection, all pigs were subjected to pharmacological euthanasia via an i.m. injection of Ketamine 350 mg/100 kg b.w., Stresnil 200 mg/100 kg b.w., Sedazin 30 mg/100 kg b.w. and Morbital (26.7 mg/mL) 0.3–0.6 mL/kg b.w. (Piwet, Puławy, Poland), intravenously. 

Immediately after euthanasia, pancreatic samples from standard locations (head, body, tail) were snap-frozen in liquid nitrogen. Portions of pancreatic tissue were also immediately fixed in phosphate-buffered formaldehyde (4% *w*/*v*). Sections of duodenum and jejunum were carefully dissected, opened at the mesentery and cleaned with saline, and the duodenal and ileal mucosa were then scraped using a glass slide and snap-frozen in liquid nitrogen. All samples were collected within 5 to 15 min after the death of the animal.

### 4.3. USG Pancreas Examination 

Ultrasound examinations of the pigs’ pancreas were performed on day 0, 7, 14, 21, 28, 35, 42, and 49, using an Aloka PS2 scanner equipped with a 6 MHz linear array transducer (Hitachi-Aloka Medical Ltd., Tokyo, Japan). The probe was placed on the left side, in the intercostal space, between the 12th and 13th rib, parallel to the course of the ribs. The scans were saved in DICOM format for further detailed analysis. Computer-assisted analysis of the scans was performed using ImageJ Fiji software (version 1.53t) [91]. The area of interest was marked using the freehand selection tool; then, analysis of the main echotextural parameters of the tissue was performed (mean pixel intensity, pixel heterogeneity) [92]. The USG examination on day 35 was possible because the team responsible for USG analysis was not subjected to COVID-19 restrictions. 

Analysis of the scans was carried out by a physician, working in a hospital, who specialized in radiology.

### 4.4. Inflammatory Parameters, Serum Nutritional and Other Basal Biochemical Parameters

Blood serum concentrations of C-reactive protein (CRP), interleukin 1 beta (IL-1- beta), interleukin 6 (IL-6), interleukin 10 (IL-10) and tumor necrosis factor alpha (TNF-alpha) were determined using commercial pig-specific enzyme-linked immunosorbent assay (ELISA) kits (#QY-E30238, Qayee-bio, Shanghai, China; #E0116Po BT-Lab, Shanghai, China; #E0122Po, #EP0086, #EP0159, BT-Lab, Shanghai, China, respectively). Blood serum total superoxidase dismutase (T-SOD) activity and the concentration of reduced glutathione (GSH) were determined using commercial assay kits (#E-BC-K020 m and #E-BC-K030-M, respectively, Elabscience, Houston, TX, USA). All assays were performed in two technical replicates according to the manufacturers’ protocols, using a Benchmark Plus microplate spectrophotometer (Bio-Rad Laboratories, Inc., Hercules, CA, USA). The intra-assay CV for all assays was below 8%. Blood serum was analyzed for lactate dehydrogenase (LDH) and gamma-glutamyl transpeptidase (GGTP), using an automatic biochemistry analyzer (Mindray BS-120, Bio-Medical Electronics, Shenzhen, China) and the respective commercial, ready-to-use tests (Alpha Diagnostics, Warsaw, Poland). Blood serum concentrations of D-lactate, a marker of intestine damage and an indicator of the permeability of the intestinal mucous membrane, was determined using a commercial D-lactate assay kit (#700520, Cayman Chemical, Ann Arbor, MI, USA).

The concentrations of IL-1-beta, IL-6, TNF-alpha and GSH in homogenized pancreatic samples that were chilled in PBS were determined using the same ELISA kits as those for blood serum analyses. 

To assess nutritional status, total cholesterol (TCHOL, mmol/L) and serum albumin (ALB, g/L) were determined in blood serum. Other biochemical parameters that were determined included: low-density lipoprotein (LDL, mmol/L), high-density lipoprotein (HDL, mmol/L), triacylglycerol (TG, mmol/L), total protein (TPROT, mmol/L), urea (UREA, mmol/L), alanine aminotransferase (ALT, U/L), aspartate aminotransferase (AST, U/L). All the analyses were performed using an automatic biochemistry analyzer (Mindray BS-120, Bio-Medical Electronics, Shenzhen, China) and the respective commercial ready-to-use tests (Alfa Diagnostics, Warsaw, Poland). All analyses were verified with the use of multiparametric control serum (Alfa Diagnostics, Warsaw, Poland).

### 4.5. Pancreas Histopathology 

The formaldehyde-fixed pancreas tissue samples were further processed to 4 µm paraffin sections. For general histopathological assessment of chronic pancreatitis, tissue sections were stained with hematoxylin and eosin and examined under a light microscope by a pathologist blinded to the treatment.

### 4.6. RT-qPCR Analysis of mRNA Expression of Genes Encoding Intestinal Barrier Proteins 

Total RNA was extracted from mucosal scrapings using the PureLink RNA Mini Kit (Invitrogen, Waltham, MA, USA), according to manufacturer’s instructions. The isolated RNA was treated with DNase I (PureLink DNase Set; Invitrogen, Waltham, MA, USA) to remove genomic DNA. The NanoDrop 2000 spectrophotometer (Thermo Fisher Scientific, Wilmington, DE, USA) was used to quantify the total RNA concentration and possible protein/chemical contamination, while the integrity of total RNA was evaluated via 2% agarose gel electrophoresis. A total of 250 ng of the total RNA was transcribed into cDNA using High-Capacity RNA-to-cDNA kit (Applied Biosystems, Walthman, MA, USA). The cDNA was subjected to RT-qPCR for examination of *CLDN1* and *CDH1* genes, encoding tight junction protein Claudin-1 and adherens junction protein, E-cadherin, respectively. To avoid DNA amplification, the primers were localized in different exons, and the primer sequences were as follows:

*CLDN1*: forward primer 5′-GGCAGATCCAGTGCAAAGTC-3′ (exon 1) and reverse primer 5′-CTGCACCTCATCATCTTCCA-3′ (exon 2) (product size 164 bp; efficiency 1.97; GenBank NM_001244539.1).

*CDH1*: forward primer 5′-CCTGCCAATCCTGATGAAAT-3′ (exon 15) and reverse primer 5′-GGAGTTCAGGGAGCTCAGA-3′ (exon 16) (product size 150 bp; efficiency 1.90; GenBank NM_001163060.1).

Primers were designed in Primer3web (https://primer3.ut.ee, accessed on 14 February 2023) and synthesized by Genomed (Genomed, Warszawa, Poland). RT-qPCR analysis was performed using RT PCR Mix SYBR Green (A&A Biotechnology, Gdańsk, Poland) on QuantStudio 7 Flex (Applied Biosystems, Waltham, MA, USA). Two genes, *RPS29* and *RPL27*, were used as an endogenous control, the expression level of which was stable across all tested tissues types and experimental conditions [93,94]. Reactions for each of the samples were carried out with three technical replicates. The amplicon’s efficiency was calculated based on slope coefficient of the standard curve from serial dilution of pooled cDNA. The relative expression of *CDH1* and *CLDN1* genes was calculated using the −ddCT method [95].

### 4.7. Western Blot Analysis

Total proteins from mucosal scrapings were extracted via ice-cold RIPA lysis buffer containing PMSF (PMSF-RO, Sigma-Aldrich Merck KGaA, Darmstadt, Germany) and protease inhibitor cocktail (S8820, Sigma-Aldrich Merck KGaA, Darmstadt, Germany). The protein concentration in supernatants was estimated using the BCA method (Pierce BCA Protein Assay Kit, Thermo Fisher Scientific, Wilmington, DE, USA). All obtained supernatants were aliquoted and stored at −86 °C.

Equal amounts of protein were separated by 12% SDS-PAGE and then transferred to PVDF membranes (Immobilon-P, Sigma-Aldrich, St. Louis, MO, USA). Blots were blocked in 5% non-fat dry milk in TBS and incubated overnight at 4 °C to an appropriate primary antibody: E-Cadherin Rb mAb (24E10, Cell Signaling, Danvers, MA, USA, dilution of 1:1000) and Caludin-1 Rb pAb (51-9000, Invitrogen, Waltham, MA, USA, dilution of 1:1000). Alkaline phosphatase-conjugated goat anti-rabbit IgG H&L (ab97048, Abcam, Cambridge, UK, dilution of 1:30,000) was used as a secondary antibody. Immunoreactive proteins were detected using standard alkaline phosphatase visualization procedure in NBT/BCIP (11681451001, Roche, Basel, Switzerland) for subsequent detection. An anti-β-actin antibody (AF7018, Affinity Biosciences Jiangsu, China, dilution of 1:10,000) was used as the loading control. Dried membranes were scanned with Perfection V850 Pro scanner (EPSON, Suwa, Japan). The semi-quantitative, normalized to their corresponding β-actin bands, densitometrical analysis of intensity of protein bands was performed using ImageJ software (version 1.51k) [91]. Uncropped original western blot membranes of E-Cadherin and Caludin-1 can be found in Supplementary Figure S1.

### 4.8. Statistical Analysis 

Statistical software G*Power (version 3.1.9.6) was used to determine the sample size that ensures statistical significance at *p* < 0.05 and a power of 0.8. A repeated measures analysis of variance (ANOVA) with the applied time as the main effect was used. The differences between the terms of sampling within the same treatment were evaluated using Tukey’s HSD post hoc test. Additionally, to determine the significance of the differences between the control and the cerulein-injected group within the same time point, one-way ANOVA, followed by Tukey’s HSD post hoc test, was performed. The normality of the data was checked using the Shapiro–Wilk test (*p* < 0.05). The statistical analyses were performed using Statistica software (Tibco Software Inc., Palo Alto, CA, USA). 

## 5. Conclusions

Based on the current results, we can conclude that repetitive cerulein injections in growing pigs led to CP over time, as evidenced by the increase in pro-inflammatory cytokines in blood serum, such as IL-6, IL-1-beta, and TNF-alpha. The increase in the inflammatory indicators (CRP, LDH, GGTP, SOD, and GSH) was also observed following cerulein injections, and CP was confirmed by USG examination. Furthermore, CP resulted in the increase in IL-1-beta, IL-6, TNF-alpha, and GSH in jejunal mucosa and pancreatic tissue. The inflammatory process in the intestine was linked to the impaired intestinal barrier, which was confirmed by the increase in D-lactate and the decreased expression of Claudin-1 and its gene in the duodenum.

## Data Availability

The data presented in this study are available on request from the corresponding author.

## Supplementary Materials

The supporting information can be downloaded at: https://www.mdpi.com/article/10.3390/ijms24097715/s1.

## Abbreviations

ALB—albumin; ALT—alanine aminotransferase; AP—acute pancreatitis; AST—aspartate aminotransferase; BW—body weight; CER-pig—the group injected with cerulein; CP—chronic pancreatitis; C-pig—the control group; CRP—C-reactive protein; GGTP—gamma-glutamyl transpeptidase; GSH—reduced glutathione; HDL—high-density lipoprotein; IL-1-beta—interleukin 1-beta; IL-6—interleukin 6; LDH—lactate dehydrogenase; LDL—low-density lipoprotein; MPH—mean pixel heterogeneity; MPI—mean pixel intensity; SOD—superoxidase dismutase; TCHOL—total cholesterol; TG—triacylglycerol; TNF-alpha—tumor necrosis factor alpha; TPRT—total protein; USG—ultrasonography.
