# Supplementary material for: Repetitive Cerulein-Induced Chronic Pancreatitis in Growing Pigs—A Pilot Study
Source: Int J Mol Sci. 2023 Apr 23;24(9):7715. doi: 10.3390/ijms24097715 (PMC10177971; doi:10.3390/ijms24097715)
Supplement: Supplementary file 1 [file ijms-24-07715-s001.zip › ijms-2314783-supplementary.pdf]

Repetitive Cerulein-Induced Chronic Pancreatitis in Growing Pigs—A Pilot Study

Ewa Tomaszewska, Małgorzata Świątkiewicz, Siemowit Muszyński, Janine Donaldson, Katarzyna Ropka-Molik, Marcin B. Arciszewski, Maciej Murawski, Tomasz Schwarz, Piotr Dobrowolski, Sylwia Szymańczyk, Sławomir Dresler, Joanna Bonior

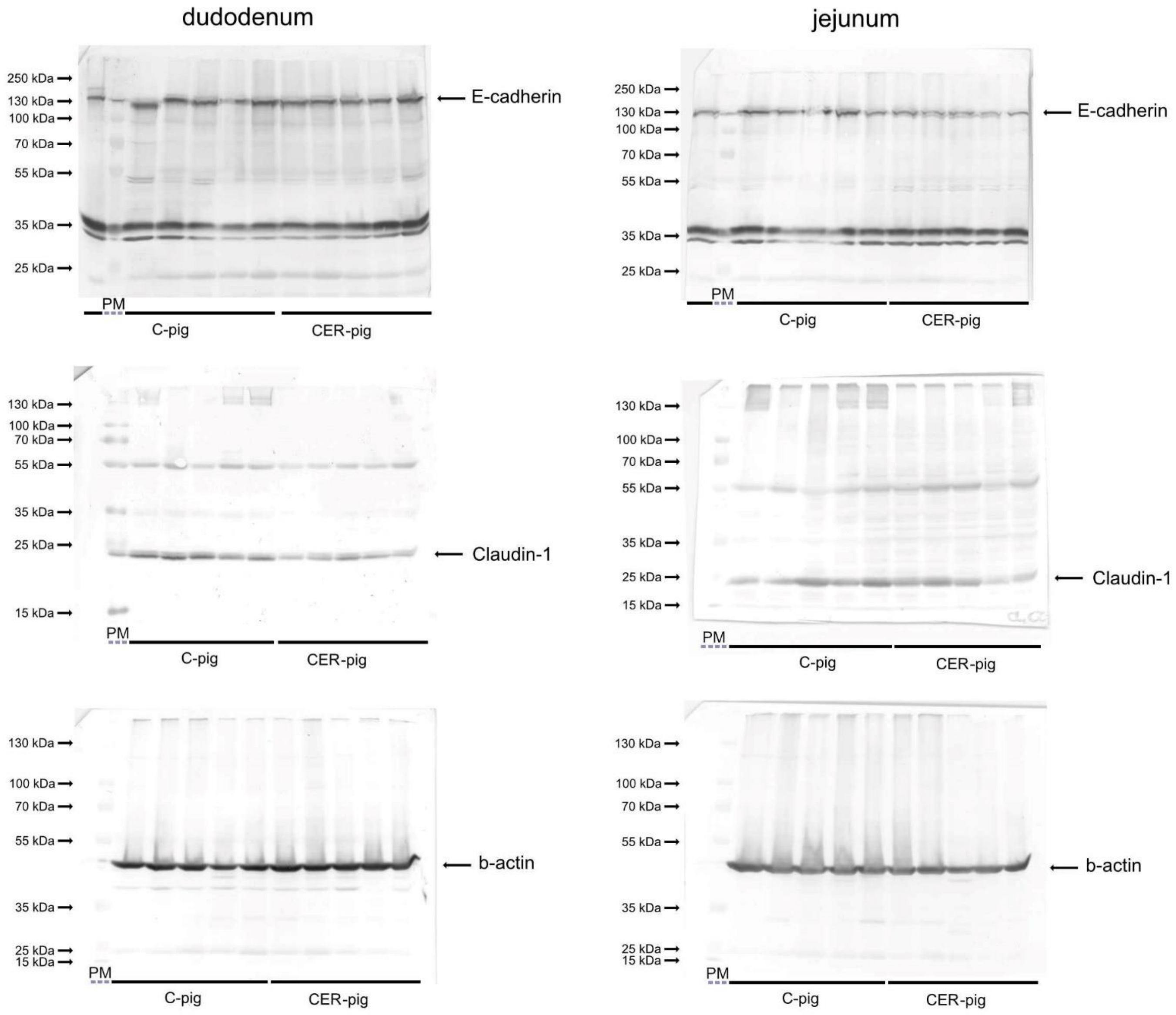

Supplementary Figure S1. Uncropped original Western blot membranes of E-Cadherin and Caludin-1 and b-actin as loading control (corresponding to Figure 7b and 7e in the main article).
